# Factors associated with public awareness of the relationship between alcohol use and breast cancer risk

**DOI:** 10.1186/s12889-023-15455-8

**Published:** 2023-03-28

**Authors:** Anne Doyle, Claire O’Dwyer, Deirdre Mongan, Seán R. Millar, Brian Galvin

**Affiliations:** 1 Health Research Board, Grattan House 67–72 Lower Mount Street, Dublin, Ireland; 2 School of Public Health, University College Cork, 4th Floor, Western Gateway Building, Cork, Ireland

**Keywords:** Alcohol, Cancer, Public, Awareness

## Abstract

**Background:**

Public awareness of the carcinogenic effects of alcohol is low, particularly the association between alcohol use and the risk of developing breast cancer. Breast cancer is the third most common cancer in Ireland and alcohol use remains high. This study examined factors related to awareness of the association between alcohol use and breast cancer risk.

**Methods:**

Using data from Wave 2 of the national Healthy Ireland Survey, a representative sample of 7,498 Irish adults aged 15 + years, descriptive and logistic regression analyses were conducted to investigate relationships between demographic characteristics, type of drinker and awareness of breast cancer risk.

**Results:**

A low level of awareness of the risk of alcohol use (drinking more than the recommended low–risk limit) associated with breast cancer was found, with just 21% of respondents correctly identifying the relationship. Multivariable regression analyses found that factors most strongly associated with awareness were sex (female), middle age (45—54 years) and higher educational levels.

**Conclusion:**

As breast cancer is a prevalent disease among women in Ireland, it is essential that the public, in particular women who drink, are made aware of this association. Public health messages that highlight the health risks associated with alcohol use, and which target individuals with lower educational levels, are warranted.

## Introduction

In 2019, alcohol use was the ninth leading risk factor attributable to deaths and disability–adjusted life–years (DALYs) in the world. For those aged 25—49 years, it was the leading risk factor, and second highest for those aged 10—24 years. Globally, alcohol use was attributable to 0.374 million (0.298–0.461) deaths in females [1]. The association between alcohol use and risk of seven cancer types; liver, oesophagus, larynx, upper throat, mouth, bowel and female breast has been well recognised in the literature [2–16]. However, despite these well– and long–established relationships, public knowledge remains poor, especially for the risk of female breast cancer [17–22].

In 2020, it was estimated that globally, approximately 4.1% of all new cases of cancer were attributable to alcohol use and that after cancers of the oesophagus and the liver, female breast cancer was the third most common cancer attributable to alcohol [11]. A 2008 study involving 363,988 participants from eight European countries taking part in the European Prospective Investigation into Cancer and Nutrition (EPIC) study examined alcohol use and incidence of cancer. Alcohol use, past and current, was obtained through a questionnaire and incidence of cancer was acquired through cancer registers, self-reports, health insurance records or death certificates. The research found that exceeding low–risk drinking guidelines was a key risk factor for alcohol attributable cancers [13]. However, since the publication of that study, the World Health Organization (WHO) have advised that there is no safe level of alcohol consumption and that even low levels of alcohol use can have harmful consequences [16], with moderate drinking (< 20 g per day) contributing to one in seven alcohol–attributable cancer cases [11]. A prospective study of 105,986 women showed even lower levels of alcohol use to be harmful; consuming ½ —1 standard drink per day (5—10 g) was associated with breast cancer and the risk was found to increase with episodes of binge drinking [5]. Another study (1,508 cases and 1,556 controls) found that consuming 15—30 g per day was associated with a 35% increase in breast cancer risk [23].

In Ireland, breast cancer is the third most commonly occurring cancer (the most common cancer among women) with on average 3,700 new cases diagnosed annually [24]. In 2022, the Irish Health Service Executive (HSE) estimated that alcohol is attributed to at least 260 of these breast cancers [25]. Although mortality rates are declining in Ireland, there are approximately 690 breast cancer deaths annually [26]. In addition, hospital discharge data demonstrate that in 2017 there were 10,667 discharges from Irish hospitals due to alcohol–related cancers, most commonly due to breast cancer discharges (28.8% of alcohol–related cancer discharges), oesophageal tract cancer (22.8%) and oropharyngeal cancer (23.0%) [27].

Concerningly, per capita alcohol consumption in Ireland was the eleventh highest of all Organisation for Economic Co–operation and Development (OECD) countries at 10.8 L in 2019 [28]. Healthy Ireland Survey data in 2018 show that 72% of women (aged 15 years and over) drank alcohol in the past twelve months and of those, just under one–half indicated weekly alcohol use (48%), an increase compared to survey data collected in 2017 (46%) [29, 30]. Binge, or heavy episodic drinking (HED) (defined as consuming 60 g of ethanol, or six standard drinks on a single occasion) remained unchanged among Irish adults between 2016 and 2018; 37% of adult drinkers reported binge drinking on a typical drinking occasion, more commonly noted among males (54%) than females (19%) in 2018 (55% of males and 18% of females in 2016) [31, 32]. These data highlight the need to increase awareness of the health risks associated with alcohol use in order to reduce alcohol-related morbidity and mortality.

Studies from the United Kingdom (UK) and the United States indicate that public awareness of the link between alcohol use and risk of developing breast cancer is low [19, 20]. Previous Irish research has shown that, with the exception of liver disease, awareness of the link between alcohol use and five health conditions (liver disease, pancreatitis, high blood pressure, female breast cancer and bowel cancer) (all of which are associated with alcohol use) is also low, with the fewest respondents aware of the breast cancer link [22]. Given that two in every 10 female drinkers in Ireland engage in hazardous drinking on a typical drinking occasion, and that breast cancer is the most common cancer among women in Ireland, the aim of this study was to investigate the level of public knowledge of the association between alcohol use and risk of developing breast cancer, and also to identify characteristics related to awareness, using a representative random sample of the Irish population.

## Methods

### Sampling and study population

Data were generated from Wave 2 of the Healthy Ireland Survey, a nationally representative cross–sectional survey of adults aged 15 years or older in Ireland. The lead author was granted access to the full dataset for the purposes of this study. The sampling frame used the An Post/Ordnance Survey Ireland GeoDirectory, a complete record of addresses (residential and commercial) in the Republic of Ireland. Electoral Division clusters were identified as primary sampling units and clusters with less than 500 addresses were combined with adjoining districts and a two–stage equal–probability sample of addresses was drawn. Stratification of the sample was by region and then by relevant variables and 686 clusters were selected and twenty addresses were then selected systematically from the list resulting in 13,720 addresses. One eligible individual aged 15 years or older in each household was selected to participate. Once respondents had consented to participate, face to face computer–assisted interviews were carried out in respondents’ own homes, with a self–completed questionnaire on sexual health. A total of 7,498 respondents took part in Wave 2 of the survey, with interviews completed between September 2015 and May 2016, achieving a 59.9% response rate. The survey included questions on a variety of topics including smoking, alcohol use, diet and nutrition, physical activity, sexual health, general health, health service utilisation and mental health. The final sample from Wave 2 was weighted by sex, age, education, region and working status to maximise its representativeness of the general population. Ethical approval was provided by the Research Ethics Committee at the Royal College of Physicians of Ireland. More details on the sample and the sampling process can be found in the Healthy Ireland Wave 2 technical report [33].

### Measures

Healthy Ireland respondents were given a showcard with a number of conditions listed (liver disease, pancreatitis, stomach ulcers, high blood pressure, skin cancer, bowel cancer and female breast cancer) and were asked to indicate which (or all) of the conditions they perceived they were at increased risk of developing by drinking more than recommended low–risk number of standard drinks in a week. The concept of a standard drink was explained to respondents, and they were presented with showcards with pictorial examples of standard drinks. According to the Health Service Executive (HSE) low–risk guidelines, women should consume no more than 11 standard drinks in a week and men no more than 17 standard drinks per week, spread out over the course of the week with at least two alcohol–free days, however this was not explained to respondents [34].

### Sociodemographic characteristics

Demographic information available included age, sex, marital status, deprivation decile, region of residence, employment status and education. Region of residence was ascertained by the address of the respondent and was grouped into Irish provinces: Munster, Connacht/Ulster (combined), Leinster (excluding Dublin) and Dublin as a separate district. The International Standard Classification of Education (ISCED) [35] was used to measure educational attainment. The ISCED has nine levels ranging from 0 to 8: ISCED 0 = early childhood education, ISCED 1 = primary education, ISCED 2 = lower secondary education, ISCED 3 = upper secondary education, ISCED 4 = post-secondary non-tertiary education, ISCED 5 = short-cycle tertiary education, ISCED 6 = Bachelor’s degree or equivalent tertiary education level, ISCED 7 = Master’s degree or equivalent tertiary education level, ISCED 8 = Doctoral degree or equivalent tertiary education level. For the purposes of this study, the nine levels were collapsed into three categories: no qualifications (ISCED levels 0—2), below degree level (ISCED levels 3–5), and degree level or above (ISCED levels 6—8). Deprivation level is based on the Irish Pobal HP deprivation index developed by Haase and Pratschke measuring the relative affluence or deprivation of a geographical area using data compiled from various censuses [36]. A score is given to the area based on a national average of 0 and ranging from − 35 (being the most deprived) to + 35 (being the least deprived). For the purposes of this study, these data are presented in three categories from most deprived (1) to least deprived (3). To ascertain employment status, respondents were asked how they would define their current situation with regard to work and asked to choose from a list of nine options displayed on a showcard. These nine options were collapsed into six categories: employed (working for payment or profit), unemployed (unemployed having lost or given up previous job, actively looking for work after voluntary interruption of working life for personal or domestic reasons, looking for first regular job), student (student or pupil), home duties (engaged in home duties, retired (retired from employment) and other (unable to work due to permanent sickness or disability, other).

### Alcohol use

Alcohol use was measured via standardised frequency and consumption questions. A showcard listing types of alcohol (for example, beer, cider, wine, cream liqueurs etc., along with some brand examples) was shown to respondents and they were asked to indicate if they had ever consumed any of the products listed (Yes/Never/Have only had a few sips of alcohol in my lifetime/Don’t know/Refused). Respondents who indicated that they had consumed alcohol in their lifetime were shown a further showcard with options of frequency of alcohol use in the last 12 months, from ‘daily’ through to ‘once a year’. When determining frequency of alcohol use, the type of alcohol consumed was not distinguished. Non–drinkers were defined as those who had not consumed alcohol in the last 12 months. Drinker status was based on WHO’s Alcohol Use Disorders Identification Test–Concise (AUDIT–C) responses; those with a score less than five were considered low–risk drinkers and those with a score five or greater were considered hazardous drinkers.

### Analysis

Descriptive characteristics and differences in factors according to knowledge (or lack of knowledge) of the link between alcohol consumption and breast cancer were analysed using cross–tabulation and statistical significance was assessed using Pearson *χ*^2^ tests. Variables identified as being statistically significant were entered into a multivariable logistic regression model which was used to estimate odds ratios (ORs) of being aware of the risk and 95% confidence intervals (CI). This model included sex, age group, education, employment status, deprivation category, marital status and drinker status. Data analysis was conducted using Stata SE Version 15.1 (Stata Corporation, College Station, TX, USA) for Windows. For all analyses, a P value of less than 0.05 was considered to indicate statistical significance. Data have been weighted to account for differential response rates.

## Results

### Sample description

Table 1 describes the drinking patterns of respondents with valid responses for alcohol use (n = 7,434). Just over one–half of respondents were female (51.2%) and 23.9% had a third-level qualification, 42.0% had below degree level and 34.2% had no formal educational qualifications. Over one–half of respondents were in employment (53.3%). Level of deprivation was grouped from the most deprived (32.3%) to the least deprived (27.3%) (2.0% missing) and over one–half of the respondents were married or in a civil partnership (55.7%).

**Table 1:** Sociodemographic characteristics and patterns of alcohol use in the population (N = 7,434)

Characteristic	N (%)	Non–drinkers N (%)	AUDIT–C < 5 N (%)	AUDIT–C 5 + N (%)	P value
**All**	7434 (100)	1871 (25.2)	2653 (35.7)	2911 (39.2)	
**Sex**					< 0.001
Male	3628 (48.8)	798 (22.0)	850 (23.4)	1979 (54.6)	
Female	3806 (51.2)	1073 (28.2)	1802 (47.3)	931 (24.5)	
**Age group**					< 0.001
15–24	1078 (14.5)	365 (33.9)	250 (23.2)	463 (42.9)	
25–34	1342 (18.1)	233 (17.4)	436 (32.5)	673 (50.1)	
35–44	1483 (19.9)	266 (17.9)	593 (40.0)	625 (42.1)	
45–54	1264 (17.0)	254 (20.1)	484 (38.3)	527 (41.7)	
55–64	1015 (13.7)	231 (22.7)	403 (39.7)	382 (37.6)	
65+	1252 (16.8)	522 (41.7)	487 (38.9)	242 (19.4)	
**Education**					< 0.001
No qualifications	2539 (34.2)	994 (39.2)	763 (30.0)	782 (30.8)	
Below degree	3121 (42.0)	613 (19.6)	1129 (36.2)	1379 (44.2)	
Degree or above	1774 (23.9)	264 (14.9)	760 (42.9)	750 (42.3)	
**Employment status**					< 0.001
Employed	3960 (53.3)	639 (16.1)	1441 (36.4)	1880 (47.5)	
Unemployed	457 (6.1)	97 (21.3)	119 (26.0)	241 (52.7)	
Student	855 (11.5)	336 (39.3)	229 (26.8)	290 (33.9)	
Home duties	938 (12.6)	345 (36.7)	428 (45.6)	166 (17.7)	
Retired	898 (12.1)	329 (36.7)	349 (38.8)	220 (24.5)	
Other	326 (4.4)	125 (38.3)	86 (26.6)	114 (35.1)	
**Region**					< 0.001
Dublin	2124 (28.6)	495 (23.3)	736 (34.7)	892 (42.0)	
Rest of Leinster	1980 (26.6)	448 (22.6)	711 (35.9)	821 (41.5)	
Munster	2030 (27.3)	550 (27.1)	733 (36.1)	747 (36.8)	
Connacht/Ulster	1300 (17.5)	377 (29.0)	471 (36.3)	451 (34.7)	
**Deprivation category**					< 0.001
1 (most deprived)	2377 (32.3)	684 (28.8)	772 (32.5)	920 (38.7)	
2	2966 (40.3)	746 (25.1)	1078 (36.3)	1143 (38.5)	
3 (least deprived)	2010 (27.3)	426 (21.2)	770 (38.3)	815 (40.5)	
**Marital status**					< 0.001
Single/never married	2535 (34.1)	680 (26.8)	676 (26.7)	1179 (46.5)	
Married/civil partnership	4139 (55.7)	918 (22.2)	1692 (40.9)	1529 (36.9)	
Widowed/separated/divorced	760 (10.2)	272 (35.8)	285 (37.5)	203 (26.7)	

Just over one–quarter of the sample were non–drinkers (25.2%). Non–drinkers were predominantly female, married or in a civil partnership, with no formal education and in the older age group (aged 65 years and over). Those who scored five or more on the AUDIT–C represented 35% of the sample and their drinking patterns indicated that they were potentially hazardous drinkers. Males were more likely than females to be hazardous drinkers as were those who were educated to degree level.

### Awareness of the relationship between alcohol use and breast cancer

Respondents’ knowledge of the link between alcohol consumption and breast cancer risk was found to be low, with just 21.2% of all Healthy Ireland respondents, representing the Irish population, correctly identifying the association. Table 2 presents the demographic characteristics of respondents in relation to their knowledge. Females were significantly more aware of the association than males (26.7% compared to 15.4%). Age was significantly associated with knowledge of the link between alcohol use and breast cancer, with highest awareness among those in the 45—54 years age bracket (26.5%), while the youngest age group (15—24 years) (12.5%) reported the lowest level of awareness.

**Table 2:** Respondents’ knowledge of the risk associated between drinking more than the recommended low–risk number of standard drinks in a week and breast cancer (bivariate association) (N = 7,498) (All respondents)

Characteristic	Total	Breast cancer		P value
		Aware of risk associated with alcohol use N (%)	Not aware of risk associated with alcohol use N (%)	
**Sex**				< 0.001
Male	3665 (48.9)	566 (15.4)	3100 (84.6)	
Female	3833 (51.1)	1022 (26.7)	2811 (73.3)	
**Age group**				< 0.001
15–24	1082 (14.4)	135 (12.5)	947 (87.5)	
25–34	1364 (18.2)	288 (21.1	1077 (78.9)	
35–44	1498 (20.0)	350 (23.4)	1148 (76.6)	
45–54	1275 (17.0)	338 (26.5)	937 (73.5)	
55–64	1019 (13.6)	231 (22.7)	788 (77.3)	
65+	1259 (16.8)	245 (19.5)	1014 (80.5)	
**Education**				
No qualifications	2560 (34.1)	404 (15.8)	2155 (84.2)	< 0.001
Below degree	3156 (42.1)	659 (20.9)	2497 (79.1)	
Degree or above	1782 (23.8)	524 (29.4)	1258 (70.6)	
**Employment status**				
Employed	3991 (53.2)	938 (23.5)	3053 (76.5)	< 0.001
Unemployed	468 (6.2)	77 (16.6)	390 (83.4)	
Student	858 (11.4)	95 (11.1)	763 (88.9)	
Home duties	946 (12.6)	249 (26.3)	697 (73.7)	
Retired	904 (12.1)	168 (18.6)	736 (81.4)	
Other	330 (4.4)	59 (17.9)	271 (82.1)	
**Region**				
Dublin	2142 (28.6)	443 (20.7)	1699 (79.3)	0.073
Rest of Leinster	2000 (26.7)	428 (21.4)	1572 (78.6)	
Munster	2043 (27.2)	403 (19.7)	1641 (80.3)	
Connacht/Ulster	1313 (17.5)	314 (23.9)	999 (76.1)	
**Deprivation category**				
1 (most deprived)	2405 (32.4)	462 (19.2)	1943 (80.8)	0.022
2	2989 (40.3)	646 (21.6)	2343 (78.4)	
3 (least deprived)	2022 (27.3)	466 (23.0)	1557 (77.0)	
**Marital status**				
Single/never married	2551 (34.0)	435 (17.0)	2117 (83.0)	< 0.001
Married/civil partnership	4178 (55.7)	1006 (24.1)	3172 (75.9)	
Widowed/separated/divorced	769 (10.3)	147 (19.1)	622 (80.9)	
**Drinker status**				0.039
Non–drinker	1871 (25.2)	391 (20.9)	1480 (79.1)	
AUDIT–C < 5	2653 (35.7)	608 (22.9)	2045 (77.1)	
AUDIT–C 5+	2911 (39.2)	574 (19.7)	2337 (80.3)	

There was a significant linear association between increasing educational attainment and greater level of awareness; those educated to degree level or higher were more likely to be aware of the risk (29.4%) compared to those with no qualifications (15.8%). Respondents who were engaged in home duties (26.3%) and those in employment (23.5%) reported a greater level of awareness. Subjects who were married or in a civil partnership were also more likely to be aware of the risk (24.1%) compared to those who were single or never married (17.0%), as were those in the least deprived deprivation category (23.0%) compared to those in the most deprived (19.2%).

Awareness of the association between alcohol use and breast cancer was found to be higher among those who were classified as lower risk drinkers (indicated by a score less than five in the AUDIT–C) (22.9%) compared to hazardous drinkers (indicated by a score of five or greater in the AUDIT–C) (19.7%), although non–drinkers showed an almost equally low level of awareness (20.9%). There was no significant association between geographical location of respondents and knowledge, with those living in the Dublin region and those outside the Dublin region reporting similar low levels of awareness.

Multivariable logistic regression analysis demonstrated that females were almost twice as likely than males to be aware of the association between alcohol use and risk of developing breast cancer (OR = 1.99, 95% CI: 1.71–2.32, p < 0.001). Those aged 45—54 years were significantly more likely to be informed when compared to those aged 15—24 years (OR = 1.71, 95% CI: 1.10–2.66, p = 0.017) (Table 3). Participants who were educated to degree level or higher were twice as likely (OR = 1.99, 95% CI: 1.60–2.47, p < 0.001) than those with no qualifications to be aware of the association and those who had below degree level qualifications were also more likely than those without qualifications to be aware of the association (OR = 1.28, 95% CI: 1.06–1.54, p = 0.01). Respondents who scored less than five on the AUDIT-C were less likely to be aware of the association compared to non-drinkers (OR = 0.84, 95% CI: 0.71–1.00, p = 0.045).

**Table 3:** Multivariable regression results for factors associated with awareness of the relationship between alcohol use and breast cancer risk

Characteristic	OR	95% CI	P value
**Sex**			
Male	1		
Female	1.99	1.71–2.32	< 0.001
**Age group**			
15–24	1		
25–34	1.12	0.73–1.74	0.598
35–44	1.30	0.84–2.01	0.232
45–54	1.71	1.10–2.66	0.017
55–64	1.53	0.97–2.41	0.066
65+	1.47	0.89–2.41	0.131
**Education**			
No qualifications	1		
Below degree	1.28	1.06–1.54	0.01
Degree or above	1.99	1.60–2.47	< 0.001
**Employment status**			
Working	1		
Unemployed	0.88	0.65–1.18	0.388
Student	0.65	0.41–1.03	0.064
Home duties	0.97	0.79–1.19	0.769
Retired	0.89	0.68–1.15	0.362
Other	0.88	0.60–1.29	0.518
**Deprivation category**			
1 (most deprived)	1		
2	1.08	0.92–1.27	0.332
3 (least deprived)	1.07	0.90–1.28	0.453
**Marital status**			
Single/never married	1		
Married/civil partnership	1.10	0.92–1.32	0.282
Widowed/separated/divorced	0.80	0.63–1.02	0.070
**Drinker status**			
Non–drinker	1		
AUDITC–C < 5	0.84	0.71–1.00	0.045
AUDITC–C 5+	0.95	0.79–1.15	0.593

Odds ratios (OR) and 95% confidence intervals (CI) are shown. All variables adjusted for each other

## Discussion

Using data from Wave 2 of the Healthy Ireland Survey, a nationally representative study of 7,498 respondents, our study demonstrates a lack of awareness of the carcinogenic effects of alcohol, with just one in five respondents being aware of the link between exceeding the recommended weekly low–risk alcohol guidelines and developing female breast cancer. Multivariable regression analysis found that factors most strongly associated with awareness were sex (female), middle age (45—54 years) and higher educational levels.

Almost two–fifths of respondents scored five or higher on the AUDIT–C indicating that hazardous drinking patterns are commonplace in Ireland and, although higher among males (54.6%), almost one–quarter of females scored five or more on the AUDIT–C (24.5%). Among all participants, although levels of awareness were similar across AUDIT–C categories, just 19.7% of respondents who scored five or higher on the AUDIT–C were aware of the association between drinking more than the recommended weekly low–risk alcohol guidelines and increased risk of developing breast cancer, a concern given their drinking patterns place them at higher risk of alcohol-related conditions.

The low level awareness of the association between alcohol use and risk of developing breast cancer we found among the general population in Ireland is similar to that seen elsewhere [17–21]. A UK study found that unprompted, less than one in five (17.8%) of a representative sample of 2,100 adults believed that the risk of breast cancer could increase as a result of alcohol use, with females (19.8%) being more likely than males (15.7%) to be aware of the association [19]. In the United States, just under one-quarter (24.6%) of a sample of 10,940 women aged 15—44 years were aware that alcohol consumption was a risk factor for developing breast cancer [20]. A further study in the UK found similar low levels of awareness, with just 19.5% of those attending a breast screening programme correctly identifying the link [21]. The study also found that less than one–half of the health professional staff at the service (48.5%) were aware that alcohol use is a risk factor for developing breast cancer.

Despite alcohol being classified as a Group 1 carcinogen since 1988, the poor public awareness of the association between alcohol use and breast cancer risk is concerning. The absence of health warning messages on alcohol products may account for this lack of knowledge and the provision of such labels could encourage recognition of the risks associated [37]. For drinkers to make behavioural changes in relation to their alcohol use, it is important that they are aware of the potential harmful health consequences associated with their drinking [38, 39]. The WHO recommend that alcoholic products inform consumers of the harms associated with alcohol use [40]. Previous research examining the effectiveness of health warning labelling found that such messages increase knowledge of the health–related risks associated with alcohol use, are effective in communicating information about low–risk alcohol guidelines and motivate changes in alcohol use behaviours, particularly among women [41–46].

In 2018, the Public Health (Alcohol) Bill was enacted in Ireland aiming to reduce alcohol consumption at a population level, delay alcohol initiation among schoolchildren, reduce the harms caused by alcohol use and to regulate and control the price and availability of alcohol based on the principles set out by the WHO’s ‘best buys’ to reduce alcohol–related harms [40, 47]. A majority of components of the Act have commenced to date, including structural separation of alcohol products in mixed retail outlets, minimum unit pricing, restrictions on alcohol advertising and sports sponsorship and restrictions on the sale and supply of alcohol, particularly price–based promotions, to reduce the affordability and availability of alcohol. However, a number of components have yet to be implemented, including Sect. 13 of the Act restricting the content of alcohol advertisements, Sect. 18 limiting advertising in print media and Sect. 19 which provides for a broadcast watershed on alcohol advertising.

Also not yet commenced is Sect. 12 of the Act, stipulating that all alcoholic products contain information about the calorie content, the quantity of grams of alcohol in the container (number of standard drinks) as well as displaying health warning information of the health risks associated with alcohol use, the danger of alcohol use when pregnant, details of an independent website providing public health information in relation to alcohol use, along with a specific warning informing the public of the direct link between alcohol and fatal cancers. This section of the Act also stipulates that this health information be displayed in on–licenced premises and on websites that sell alcohol products. Ireland is one of only two countries in the world (along with South Korea) that has enacted legislation mandating the display of cancer warning messages on alcoholic products and to date, to the best of our knowledge, there have not been any published literature on the outcomes of such specific labelling [48].

Knowledge of factors related to awareness of the association between alcohol use and breast cancer risk could help inform public health campaigns in raising awareness. As previous studies have observed, our data show that women are more likely than men to be aware of the association, and this is reassuring as the risk is sex specific. However, awareness among women was low, and more than one-fifth of female respondents reported drinking in a hazardous manner. As breast cancer is the most prevalent cancer among women in Ireland, it is therefore essential that women are made aware that they are increasing their risk through targeted campaigns. Age was also significantly associated with awareness; those aged 45—54 years more likely to know of the risk between alcohol use and breast cancer. Younger respondents showed poorer awareness and, along with the 25—34 years age group, represented the highest level of hazardous drinking and thus undoubtedly require a strategic awareness campaign. Also importantly, education level was significantly associated with awareness; those with lower levels of educational attainment were less likely to be aware of the association. Therefore, it is important that public health messages are strengthened to highlight the health risks associated with alcohol use and that individuals with lower levels of education are targeted [16].

As well as educating women about the association between alcohol use and breast cancer, it is important that men are informed too, in order that they may provide support and advice to female family members, friends and colleagues. Research to gain an insight into why women who are aware of the risk continue to drink, and to binge drink, is needed. Understanding drinking motivations is important to consider and may help inform effective public health messaging. Further research is also required to determine other factors which may influence awareness, such as social media campaigns, health practitioner advice and education programmes in communities and schools.

Considering the media commentary surrounding the consultation process for alcohol labelling, along with the other measures within the Public Health (Alcohol) Act that have commenced in Ireland, the harmful effects of alcohol use is a topic that has gained particular scrutiny in recent years. Due to this coverage, it is desirable that public knowledge of the carcinogenic effects of alcohol is examined again, particularly its link to breast cancer, as awareness may have increased due to this publicity. Finally, consideration should be given to revising the low-risk drinking guidelines in Ireland in view of WHO recommendations that there is no safe level of alcohol use.

### Strengths and limitations

The use of a random probability sample ensures that the findings are generalisable to the whole population. Using the AUDIT–C, an international standardised measure for classifying hazardous drinking ensures that such drinking patterns are reliably classified. However, the use of the AUDIT–C as opposed to the full AUDIT is a limitation of the study. Further limitations to consider when interpreting these findings are that despite the large and representative sample used, heavy and risky drinkers are often not well represented in such surveys [49] and that the sample was selected from private households, therefore those living in institutions, those who were homeless or members of Travelling community not living in permanent housing were excluded from participation. Also not represented were those who did not speak English as interviews were conducted in English only.

Furthermore, as the Health Ireland Survey only includes questions about alcohol use in the previous 12 months, it may be difficult for respondents to accurately remember their drinking behaviours for that time period. In addition, self–reporting bias may be a factor in responses resulting in an under–reporting of alcohol use, commonly seen in such surveys [50]. Finally, when respondents were given showcards listing health conditions and asked to identify if they were associated with drinking more than the recommended weekly low–risk alcohol guidelines, the number of standard drinks as per the guidelines were not explained although pictorial examples of standard drink sizes and commonly consumed drinks were provided.

## Conclusion

In agreement with previous studies, our data indicate that public awareness of the association between alcohol use and breast cancer risk is low. Considering alcohol use is a modifiable risk factor, increasing drinkers’ knowledge of this risk could help contribute to a decrease in hazardous drinking as well as reducing breast cancer incidence. Raising awareness among women, especially younger female drinkers, is important. In addition, health warning labels on alcohol products are needed. Public health messages that highlight the health risks associated with alcohol use, and which target individuals with lower educational levels, are also warranted.

## Data Availability

The data used and analysed for the purpose of this study are available from the corresponding author on reasonable request.
